# Supplementary material for: Are carbon emissions trading and green financial instruments synergistic? -Comprehensive quantitative research based on content analysis
Source: PLoS One. 2024 Mar 7;19(3):e0298601. doi: 10.1371/journal.pone.0298601 (PMC10919601; doi:10.1371/journal.pone.0298601)
Supplement: S1 Data — (ZIP) [file pone.0298601.s001.zip › Data/地级市Z_F工作报告.zip!/╣π╢1⁄2/╓Θ║ú╩╨/└┤╫╘╣1⁄2╓┌║┼í╛┬φ┐╦╩2╛▌═°í┐_2005.docx]

各位代表：

　　现在，我代表市人民政府向大会作政府工作报告，请予审议，并请政协各位委员和其他列席人员提出意见。

　　2004年工作回顾

　　2004年是我市改革开放和现代化建设继续取得重要进展的一年。在省委、省政府和市委的正确领导下，在市人大及其常委会、市政协的监督支持下，新一届市政府坚持以邓小平理论和“三个代表”重要思想为指导，深入贯彻党的十六大、十六届三中、四中全会和张德江同志考察珠海重要讲话精神，认真执行中央宏观调控政策，树立和落实科学发展观，按照市第五次党代会的部署，扎扎实实做好改革、发展和稳定的各项工作，全面完成市六届人大一次会议确定的工作任务，推动我市物质文明、政治文明和精神文明建设取得了新的成绩。

　　一、经济发展保持良好势头

　　贯彻中央宏观调控政策取得成效。认真开展土地市场专项治理，合理撤并工业园区。按照“有保有压”原则，优化投资结构，制造业、基础设施和社会公共事业成为投资增长的主要领域，投资保持合理增长，全年固定资产投资总额179.8亿元，增长27.5%。加强物价监控，全年消费价格总指数上升2.9％，涨幅低于全国和全省平均水平。金融机构加强信贷管理，年末全市金融机构存款余额769.7亿元，增长14.3％；贷款余额417.2亿元，与年初持平。电力部门科学调度，企业积极配合，实施错峰用电有效缓解电力供求矛盾。全年供购电总量50亿千瓦时，增长16.1％。全市经济在宏观调控中实现快速发展，全年完成生产总值546.28亿元，同比增长13.8％。其中，第一、二、三产业分别增长3.6％、15.1％和12.7％。全市预算内财政总收入56.1亿元，其中财政一般预算收入34.46亿元，按年初预算可比口径计算增长18.6％。

　　工业经济规模扩大。全年完成工业总产值1291.28亿元，增长24.4％。“工业西进”成效明显，金湾、斗门两区对全市工业增长贡献率提高。工业结构调整加快，电子信息、家用电器、精密机械制造、石油化工、电力能源等支柱产业和伟创力、格力电器、佳能、碧阳化工等优势企业对全市工业发展的支撑作用增强。工业重型化、高级化趋势明显，轻重工业比例调整为38.5：61.5。鼓励和扶持企业技术改造成效显著，高新技术产业蓬勃发展。珠海国家高新区在全国53个高新区中综合排名进入前二十名，经济增长幅度排名第二。全市经省认定的高新技术企业增加到123家。高新技术产品产值467.2亿元，增长30％，占工业总产值的36.2%。我市成为国家首批电子信息产业基地和国家火炬软件产业基地，全市软件产业产值达50亿元，增长43％。

　　农业生产稳步发展。及时果断处置高致病性禽流感疫情。在全省率先免征农业税，落实种粮直接补贴政策，财政和农村信用社支农资金投入增加。全市行政村实现“村村通水泥路”、“村村通公共汽车”，完成农村渡口改造，农村生产生活条件改善。加强农业招商，农业经贸洽谈会取得好成绩。扶持农业龙头企业发展，建成平沙万亩罗非鱼养殖基地等一批农业产业化基地，农业效益提高。全市农林牧渔业总产值31亿元，同比增长5.9％。

　　服务业显著上升。旅游业快速发展，全年接待游客总人数1273.6万人次，增长26.2％；旅游总收入103.7亿元，增长23.9%。大型旅游项目港中旅珠海海洋温泉度假区加紧建设。成功举办第五届中国航展，会展业有新发展。房地产市场升温，新开工项目和新开工面积大幅增加，房地产交易活跃。物流业畅旺，港口货物吞吐量3203.2万吨，增长29.7％。商贸市场兴旺，居民消费结构逐步升级，汽车、住房、通讯、文体、教育、娱乐消费需求高涨。全年实现社会消费品零售总额179.9亿元，增长13％。

　　区镇经济蓬勃发展。金湾区、斗门区加快推进农村工业化、城镇化和农业产业化，经济总量、财政收入增长速度高于全市平均水平。香洲区重点发展现代服务业、科技产业，产业升级趋势明显。各镇村结合自身特点，发展特色经济，镇村经济活力增强。珠海国家高新区、珠海保税区、临港工业区、横琴经济开发区、万山海洋开发试验区发挥各自功能优势，展现出良好发展前景。

　　二、改革开放迈出新步伐

　　政府自身改革加快。进行新一轮政府机构改革，单独设置市安全生产监督管理局，强化市城市管理局职能，将市旅游局由事业单位改为政府职能局，市广播电视局并入市文化局，对20个部门职能进行了调整，政府机构和职能设置更加适应经济社会发展的需要。严格执行《行政许可法》和国务院《全面推进依法行政实施纲要》，清理行政审批许可事项，取消行政许可77项。全面推行政务公开，为市民设立“现行文件阅览服务中心”，电子政务运行良好，开通“政信通”信息服务平台，市政府门户网站在全国评比中名列地级市第一名。从有利于节约、有利于工作、有利于廉政出发，稳妥推行公务用车制度改革。

　　市场体制改革深化。改革投融资体制，进一步确立企业的投资主体地位。设立市金融服务办公室，加强金融管理服务，积极防范和化解金融风险。培育、支持企业上市取得丰硕成果，华发股份、德豪润达、威尔科技、东信和平四家公司成功上市。成立市有形要素市场管理办公室，加强对产权、土地等要素市场监管，提高规范运作水平。继续整顿和规范市场经济秩序，整治旅游定点商店售卖假冒伪劣商品，严厉打击传销和变相传销、偷税漏税、逃汇骗汇等违法犯罪活动，保持打击走私高压态势。治理车辆超限超载取得初步成效。

　　国有资产管理体制和国企改革稳步推进。新组建市国有资产监督管理委员会。委托中介机构对国有企业进行全面清产核资。推行国有资产经营预算管理，开展经营效能监察，国有企业经营效益提高。市属国有及国有控股企业实现净利润约7.5亿元，增长6.5％。

　　民营经济发展加快。认真落实省、市一系列扶持民营经济发展的政策措施，强化引导服务，支持银企合作和信用担保机构发展，拓宽民营企业融资渠道。据经济普查数据显示，全市个体工商户达5.05万户，私营企业8900多户。个体私营企业注册资金250.3亿元，增长15.1％。民营企业在科技创新、外贸出口、增加就业和税收等方面贡献日益突出。

　　外向型经济快速发展。积极参加泛珠三角区域经贸洽谈会、中国-东盟贸易洽谈会和粤台经贸洽谈会等大型经贸活动，招商引资取得新成效。全市新引进外商直接投资项目472个，实际利用外资4.7亿美元，增长23.8％。对外贸易规模迅速扩大，进出口总额218.1亿美元，创历史新高。其中出口总额90.4亿美元，增长30.8%。

　　区域经济合作取得突破性进展。落实赴港澳旅游“自由行”政策，加快“大通关”建设，人员往来更加便捷，拱北口岸全年出入境人数突破6000万人次，横琴口岸客货通关量快速增长。与香港联合赴欧洲、印度开展招商活动。在湾仔等地建设CEPA概念商业街，一批港澳服务业进入珠海。珠澳跨境工业区建设取得重大进展，已完成填土工程，招商引资势头良好，首批4个项目动工建设。向国家申报设立“泛珠三角横琴经济合作区”，横琴岛开发建设迎来新机遇。加强对内招商，积极组织企业参加我省赴广西、湖南和东北三省的经贸活动，全力做好对口帮扶重庆三县工作，与内地经贸联系加强。

　　三、城市建设取得新进展

　　重大交通设施项目取得重要进展。调整充实重大项目推进工作指挥部，大力推动重大交通基础设施项目建设。配合国家、省和港澳有关方面做好港珠澳大桥前期工作，已完成项目工程可行性研究。广珠铁路被列为广东省与铁道部省部合作项目，铁路功能、线路等也完成了新的论证和协调。广珠城际快速轨道项目已获国家批准，进入动工建设阶段。江珠高速公路、粤西沿海高速公路珠海段工程进展良好。省道272线珠海段改造项目完成工程可行性研究。高栏港区两个５万吨级集装箱码头已列入国务院核准的港口建设规划，基础工程开工建设。

　　城市基础设施不断完善。加快能源建设，珠海电厂3号、4号机组扩建土建工程进展迅速，完成技改扩能的洪湾电厂即将投产发电，拱北220千伏变电站预计6月底竣工投入使用。中海油天然气接收站落户横琴。城市管道燃气项目建设进展顺利，首期工程点火投入运营，居民用户超过3000户。建设白蕉联围达标加固工程、草塘湾应急渡汛工程，城市防洪能力提高。制定“西水东调”方案，广昌应急水源工程建成通水，提高咸潮期供水保障能力。完善城市道路系统，柠溪西路、三台石路竣工通车，情侣北路建设进展良好。

　　规划和土地管理得到加强。新设立市规划局，充实规划人才队伍，完善规划决策机制，增加规划经费，加紧编制城市建设急需的各类规划，完成城市规划项目32项。严格执行国务院《关于深化改革严格土地管理的决定》，健全土地管理重大事项集体决策机制，全年召开土地管理领导小组会议16次，解决了234个土地管理遗留问题。收回、整理、储备一批土地，提高了政府对土地资源的调控能力。公开成功出让9宗土地，全年土地收入达20.4亿元。

　　城市建设管理水平提高。稳步推进城中旧村改造，确保房地产业持续健康有序发展，房地产开发整体质量提升。盘活市区繁华地段一批烂尾楼。加强建筑市场监管和调控，提高工程质量。城市管理相对集中行政处罚权工作成效显著，市政、园林、绿化、环卫、路灯管理不断改善，市容市貌更加靓丽。

　　环境保护成效显著。落实环境保护责任制，对重点工业污染源实行在线监控，加强环境综合整治。前山河清淤整治初见成效，全面实施城市污水管网改造工程，初步完成白沙河明渠整治，新建唐家、南湾、香洲二期、吉大二期污水处理厂，拱北污水处理厂三期工程投产，生活污水处理率提高。扩大红树林种植面积，加强沿海滩涂保护。城市环境综合考核取得好成绩，被推荐为国家环保模范城免检城市。

　　四、社会事业实现新发展

　　科技教育事业加快发展。科技自主创新能力逐步提高，有6项成果获得国家和省科技奖、14项获得市科技奖。全市申请专利1370件，增长38.5％。科技三项费用使用绩效得到提高。公共技术平台建设取得成效，集成电路设计服务中心建成运行。完成基础教育发展规划，农村地区教学环境得到较大改善。市实验中学高中部建成开学，中小学优质学位增加，高考取得历史最好成绩。制定《珠海市民办中小学管理规定》，民办教育有新发展。珠海城市职业技术学院建成开学，加快培养技术型、实用型人才。高等教育规模扩大，珠海大学园区全日制大学生达4.2万人。

　　社会各项事业全面发展。挖掘人文历史资源，成功举办纪念容闳系列活动。市青少年妇女儿童活动中心建成启用。4个基层文化站获省特级文化站称号。全民健身运动蓬勃开展。举办摩托车世界6小时耐力赛、国际汽联GT锦标赛、中甲足球联赛等重大体育赛事。建立突发公共卫生应急机制，传染病得到有效预防和控制，新型农村合作医疗覆盖率达到77.2％。人口与计划生育工作继续走在全省前列，香洲区被评为“全国计生优质服务区”。双拥共建成绩突出，蝉联全国和广东省“双拥模范城”称号。征兵、人防工作受到省表彰。开展经济普查，统计工作为政府决策提供了良好经济信息服务。全面编制部门预算，对财政性资金、政府投资项目加强审计监督，提高了资金使用绩效。青少年教育和保护工作得到加强。老龄工作、残疾人和慈善事业进一步开展，新闻、出版、档案、仲裁、民族、宗教、外事、侨务、气象、港澳流动渔民工作都有新进步，工会、共青团、妇联和工商联、科协、文联、侨联、社科联充分发挥作用，为社会事业协调发展作出了贡献。

　　民主法制建设不断加强。自觉接受市人大及其常委会的监督，认真执行市人大决议。全体政府组成人员向市人大常委会述职，7名政府部门负责人接受公开述职评议。重大问题通过市政协广泛征求民主党派、工商联、无党派人士和人民团体的意见。办好市人大议案、代表建议和政协提案，全年共办理答复代表建议218件、政协提案246件，花大力气办理4件未结的积案。加大普法工作力度，增强公务员的法律意识。建立行政执法责任制，受理并处理行政执法投诉43件。政府建章立制和行政复议工作得到加强。深入开展机关作风建设，规范窗口服务，继续做好重点项目督查督办工作，提供优质现场服务。加强廉政建设，预防职务犯罪，开展领导干部任期经济责任审计，从源头上预防和治理腐败。严厉打击各种违法犯罪活动，加强社会治安综合治理，对口岸地区进行重点整治，全市刑事发案下降，破案率上升，社会治安秩序良好。奖励见义勇为人员，维护社会公平与正义。

　　人民生活水平不断提高。城镇居民人均可支配收入达18347元，增长10.5％；农渔民人均纯收入6048元，增长9.62％。城乡居民储蓄存款余额408.6亿元，比上年增加14.3％。全市新增就业岗位4.8万个，城镇登记失业率为2.8％，超额完成万名农民转产就业工作目标。社保体系逐步完善，保险基金征缴率达99％。住房公积金稳健运作。建立健全社会救助体系，落实最低生活保障制度，实现应保尽保。对贫困大学生进行资助，不让一名学生因贫失学。全面清理建设领域欠薪，拖欠农民征地补偿款1.7亿元全部兑现。继续改造平沙、红旗地区危房。加强食品药品安全监督。落实安全生产责任制，开展专项整治，改善临港工业区、前山地区等重点区域的消防条件，实现省下达的安全生产事故控制指标任务，安全生产形势平稳。

　　各位代表！过去一年政府工作取得的成绩，是上级正确领导的结果，是全市人民不断开拓进取、扎实工作的结果，是中央、省驻珠机构和驻珠军警部队大力支持的结果。在这里，我代表市人民政府，向为珠海建设付出辛劳的全市人民致以崇高的敬意！向关心支持珠海发展的海内外朋友们表示衷心的感谢！

　　我们清醒地看到，珠海经济社会发展中还存在不少困难和问题。主要是：经济总量仍然偏小，发展速度与珠三角先进城市相比仍有差距；经济发展的质量和效益还不高，经济结构有待优化；财政实力较弱，处理历史遗留问题任务仍相当艰巨；发展不平衡，部分群众生活还比较困难；经济社会发展仍面临着体制性障碍，改革攻坚任务繁重；政府在社会管理和公共服务方面仍然存在一些薄弱环节；部分公务员能力和素质有待提高，工作作风尚待改进。对这些问题，我们要高度重视，并采取切实措施加以解决。

　　2005年工作安排

　　珠海发展任重道远。省委、省政府要求把珠海建设成为珠江三角洲现代化区域中心城市和我省实践科学发展观先行示范市，这是我们的奋斗目标。2005年是进一步贯彻落实科学发展观，巩固宏观调控成果，实现“十五”计划目标，衔接“十一五”发展的关键一年。做好今年的工作，意义十分重大。我们要进一步增强责任感、使命感和紧迫感，从国内外发展大势和珠海实际情况出发，抢抓机遇，迎接挑战，开拓创新，真抓实干，努力推动珠海经济社会在2005年实现更快更好发展。

　　2005年市政府工作的总体要求是：高举邓小平理论和“三个代表”重要思想伟大旗帜，学习贯彻胡锦涛总书记视察广东重要讲话精神，认真落实中央、省和市委一系列战略决策和重要工作部署，以科学发展观统领经济社会发展全局，按照“五个统筹”要求，深化改革，扩大开放，以推进新型工业化为主线，加快经济发展；抓住调整结构这个关键，提高经济质量和效益；统筹利用资源，积极化解发展中的矛盾和问题；坚持以人为本，致力改善民生，构筑社会主义和谐社会；加强环境保护，不断优化环境，增强可持续发展能力；切实加强政府自身建设，科学行政，民主行政，依法行政，扎实推动珠海“三个文明”建设，加快建设实践科学发展观先行示范市。概括起来，今年要围绕“加快发展，提高质量，化解矛盾，改善民生，优化环境，依法行政”的总体思路来做好政府各项工作。

　　今年我市经济社会发展的主要预期目标是：GDP增长13％；工业增加值增长15％；财政一般预算收入增长12％；社会消费品零售总额增长11％；固定资产投资增长14％；外贸出口增长10％；实际利用外商直接投资增长10％;常住人口自然增长率控制在7‰以下；城镇登记失业率控制在3％左右；城镇居民年人均可支配收入增长8％；农渔民年人均纯收入增长8％。

　　为实现上述目标，要做好以下工作：

　　一、抢抓机遇，加快经济发展

　　加快推进新型工业化。加快珠海发展关键在于推进新型工业化。要充分认识加快工业发展的重要性和紧迫性，把推进新型工业化作为全市经济工作的主线，依靠工业发展带旺服务业，通过工业发展反哺农业。要坚持“大办工业”，发挥市区镇各级的积极性，将经济工作的重心都放在发展工业上；要注重“办大工业”，着力引进大型项目、龙头企业，充分发挥龙头企业对产业的带动作用。要抢抓国际产业调整转移加快、国内省内产业结构调整出现积极变化等重大机遇，积极参与新一轮国际、国内和区域的产业分工，以发展先进制造业为方向，努力做大做强电子信息、电气机械、精密机械制造、计算机软件及集成电路、医药及医疗器械等现有优势产业，同时着力发挥临海、临港优势，努力争取在石油化工、电力能源、装备制造等重化工业发展方面实现更大突破。

　　抓好工业载体建设。进一步理顺体制，落实简政放权措施，市着重抓好五个经济功能区的发展，各个工业园则以区、镇为开发建设主体，放手发展。加强园区管理队伍建设，调整绩效考核办法，形成良好的激励机制。强化产业政策引导，建设产业公共服务平台和支撑体系，着力培育产业集群。加快园区基础设施配套建设，完善工业园区生活服务区配套，提高园区综合开发水平，促进园区开发与城镇建设相协调，发挥园区建设更大的带动作用。

　　突出抓好外向带动。外向带动是珠海发展的优势。要进一步优化投资环境，合理降低港口和路桥收费，设立“建设项目集中服务大厅”，完善项目服务机制。创新招商方式，提高招商引资水平。抓住龙头项目带动产业招商，特别要大力吸引世界500强企业等跨国公司的投资，注重从单个项目招商扩展到上下游配套的产业链招商，鼓励现有企业增资扩产，支持碧阳化工PTA二期等工程扩建。切实加强对内招商，发挥区位和环境等方面的优势，吸引内地大中型国有企业、上市公司、民营企业到珠海投资。积极发展总部经济。坚持以质取胜、科技兴贸，优化商品结构，积极拓展国内外市场，扩大进出口。积极引导企业扩大本地采购比例，引导加工贸易企业转型升级，提高企业增值率和对本地财政贡献率。

　　不断增强创新动力。创新是推动发展不竭的动力。继续深化市场取向的经济体制改革，充分发挥市场在资源配置中的基础性作用。加强有形要素市场管理，进一步规范产权交易中心等七个有形要素市场运作。加强金融服务，促进金融业健康发展，防范金融风险。整顿和规范市场经济秩序，严厉打击制假售假、走私贩私、商业欺诈、偷逃骗税、非法传销等不法行为。加快行业协会和中介组织发展，健全现代市场体系。推动社会信用体系建设，努力营造公开、公正、公平的市场环境。

　　积极参与区域合作。以推动建立“泛珠三角横琴经济合作区”为重点，积极参与泛珠三角区域合作。加强与珠三角周边城市在产业、科技、基础设施、环保等领域的合作，主动接受广州、深圳等中心城市的辐射。落实CEPA及其补充条款，积极吸引港澳服务业进入珠海。加快珠澳跨境工业区建设，扩大珠澳在旅游会展、跨境基础设施、口岸通关、现代物流等方面的合作。加强对台经济技术交流与合作。积极参与西部大开发和振兴东北老工业基地，实现互动发展。

　　全力推动重大基础设施建设。完善基础设施是突破珠海发展“瓶颈”的重要举措。积极配合国家、省和港澳有关方面，做好港珠澳大桥前期工作。支持配合广珠城际快速轨道珠海段建设。全力推动广珠铁路尽快复工。做好江珠高速公路、粤西沿海高速公路珠海段路网衔接工作。加快南横路、省道272线机场东段等公路建设，今年内建成通车。做好全市交通路网调整综合研究工作。加快高栏港区两个五万吨级集装箱码头建设，推进珠海港大型石化公用码头项目建设，建设洪湾港一期工程，争取年底竣工。继续完善城市水利设施，加快白蕉联围、小林联围等水利项目建设，增强防洪能力。优化配置水资源，加快“西水东调”工程前期工作。做好LNG接收站建设前期工作，争取尽快动工。跟踪服务珠海电厂和洪湾电厂扩能项目，完善电网建设，加强运行调节，保障电力供给。

　　二、调整结构，提高经济质量和效益

　　大力发展高新技术产业。增强企业科技创新能力，逐步实现从“珠海组装”到“珠海制造”、“珠海创造”的产业结构调整升级。发挥科技三项经费、技改贴息资金作用，按照重点产业优先、骨干企业优先的原则，完善技术创新服务体系，重点建设集成电路、生物医药等公共技术平台，提升企业自主创新能力。继续支持企业加快技术改造，特别是鼓励运用先进技术对传统行业进行技术改造。加强知识产权保护，完善技术成果入股、提成和科技奖励并重的激励机制，促进科技成果转化。推进信息化建设，重点扶持南方软件园和留学生创业园建设，抓好珠海金山软件股份有限公司WPS网络办公系统高技术产业化示范工程，促进计算机软件及集成电路产业加快发展，走信息化带动工业化的发展道路。

　　发展壮大内源型经济。进一步深化国有企业改革，推进国有资产结构调整和战略重组，国有资本重点向先导性、基础性、公益性领域和优势产业发展。积极引进战略合作伙伴，实现国有企业产权多元化。推进市属资产营运机构改革。加强国有企业内部管理，健全规范、高效、协调的国有资产监管体制。认真落实扶持民营经济发展的政策措施，深化投融资体制改革，落实社会投资基本建设项目登记备案制，拓宽民营资本投资领域。进一步完善信用担保体系，逐步缓解民营企业融资难问题。积极培育符合条件的民营企业上市。加强对民营企业的引导和服务，为民营企业发展营造优良环境。

　　加快发展现代服务业。认真贯彻省政府《关于加快我省服务业发展和改革的意见》，坚持生产服务业和生活服务业并举，推动我市服务业全面发展。继续推动以旅游、房地产为龙头的第三产业发展。提升现有旅游景区吸引力。加强旅游项目招商，开发海岛、温泉等特色旅游资源。支持珠海海洋温泉度假区、东澳岛休闲度假和水上娱乐等旅游项目加快建设，打造旅游文化品牌产品和旅游龙头企业。加快规划建设会展中心，积极发展会展业。继续推进城中旧村改造。加大市场调控力度，有计划地推出商用土地，引导房地产业健康有序发展。加快特色商业街区建设，积极发展连锁经营、电子商务等新型流通业态。优化消费环境，培育新的消费热点。加紧做好西部地区商业设施规划和市场培育工作，带动农村消费。加大开放力度，依托“双港”和海岛中转仓储优势，鼓励和引导现代物流业发展。

　　优化企业结构。实施名牌带动战略，落实奖励措施，培育发展一批掌握核心技术、拥有自主知识产权的名牌产品。继续培植大企业，鼓励现有龙头企业和骨干企业发展壮大，发挥大企业的“榕树效应”。培育一批“小而强、小而精、小而专”的中小企业群，引导中小企业与大型企业相互配套，延长产业链条，增强中小企业竞争力。

　　促进区域经济协调发展。香洲区要重点发展高新技术产业、资金技术密集型产业和现代服务业，积极推进城区老工业小区改造。金湾区、斗门区在壮大工业总量的同时，要加快西部地区城市化进程，适时规划建设新城镇，使工业化与城市化协调发展。万山区要在保护好资源的前提下，有重点地发展海岛旅游、中转仓储和海洋渔业。

　　三、统筹资源，积极化解发展中的矛盾和问题

　　严格管理财政资金。积极培植税源，进一步改善税收征管手段，加强非主体税及非税收入征管，依法征收土地使用税和堤围防护费，千方百计组织收入。完善激励型财政体制，发展壮大区级财政。树立过紧日子思想，坚决压缩一般性财政支出，保障重点支出。加快“金财工程”建设，通过信息化手段，强化财政收支监控。认真落实《珠海市政府投资项目管理条例》，强化工程招投标监管，严格执行政府年度投资项目计划和财政预算，完善国库集中支付和政府采购制度，增强预算执行和政府投资项目的审计监督力度，提高财政资金使用绩效。

　　珍惜使用土地资源。土地是关系珠海持续发展不可再生的宝贵资源。要认真执行国家和省关于严格土地管理的规定，珍惜和用好珠海每一寸土地。加快完成土地管理领导体制改革。健全土地储备、开发、使用、管理机制，科学调控土地资源。巩固土地市场整治成果，坚持依法管地、征地和用地。盘活存量土地，严格控制供地条件，提高产业用地产出率。依法严格保护好耕地和林地。

　　加强城市公共资源经营管理。推进政府公共资源特许经营改革，试行城市公共资源开发、使用、经营公开招标，增加公共资源收益。大力推动城市公用事业产业化发展、市场化运作、企业化经营、规范化管理进程。加强对国有资产、机关和事业单位经营性资产及收益的管理。

　　积极解决债务等突出矛盾。要以负责任的态度，积极化解发展中的矛盾和问题，尤其要正确处理发展与还债的关系。总的思路是在加快发展和壮大经济实力的基础上逐步偿还债务。既要加强与债权人的沟通，争取理解和支持；又要通过统筹资源，尽最大努力清偿债务。

　　四、改善民生，努力构建和谐社会

　　突出抓好“三农”工作。按照统筹城乡发展、以工哺农、以城带乡的新思路，加大对“三农”的支持力度。依托工业园区的发展，加快推进农村工业化、城镇化和农业产业化。加强现代农业产业园区和基地建设。加大农业基础设施建设力度，改善农业生产条件。继续调整农业产业结构和农村经济结构。引进和培育带动性强的农业龙头企业，大力扶持农产品加工和流通企业。加强动植物防疫工作和农产品质量安全管理。组建农民专业合作组织。鼓励村集体和村民发展为园区配套的物业出租等低风险的第三产业，拓宽农民增收渠道。积极扶持从事捕捞业的珠海渔民转产转业。认真做好村民委员会换届选举工作。

　　做好就业再就业工作。就业是民生之本，市区镇各级政府都要把扩大和促进就业作为重中之重的工作来抓。大力开发就业岗位，健全公共就业服务体系，积极开展“订单式”岗前技能培训和创业培训，鼓励社会力量参与农民就业培训。抓好高校毕业生、复员转业军人安置和城镇新增劳动力就业。力争全年新增就业岗位4.5万个，下岗失业人员再就业率达到60％以上。进一步加大劳动保障监察执法力度，依法维护劳动者的合法权益，营造稳定和谐的劳动关系。

　　改善困难群众生产生活。完善社会保障体系，不断扩大社会保险覆盖面，提高非公有制企业社保参保率，研究完善灵活就业人员参保办法。加快建立农村养老保障制度，进一步完善新型农村合作医疗制度。继续做好法律援助工作，积极解决城区低保无房户住房难等困难群众“四难”问题。健全城乡居民最低生活保障制度，做到应保尽保，确保城乡居民无饥寒。

　　维护社会稳定和安全。进一步强化社会治安综合治理，严密防范、严厉打击各种违法犯罪活动。开展创建“和谐平安社区”活动，抓好重点地区和“两抢两盗”等突出治安问题的整治，加强保安及综治队等各类群防群治队伍建设，进一步增强人民群众的安全感。加强普法宣传教育，引导群众以理性合法的形式来表达自身的利益诉求。认真执行国务院《信访条例》和市政府《关于维护信访秩序的通告》规定，做好信访和人民调解工作，积极预防和妥善处置群体性突发事件，营造文明法治、谅解宽容、和谐安宁的社会环境。落实《安全生产法》和安全生产责任制，加强执法队伍建设，健全监管体制，建立重大危险源、重大事故隐患监管体系，完善安全生产应急救援体系，进行道路交通安全综合整治，减少交通事故，确保全市安全生产形势稳定。进一步抓好食品药品安全监管。

　　加快各项社会事业发展。促进各级各类教育协调发展。巩固提高基础教育水平，启动金湾区、斗门区“教育强区”工程。完成市三中扩建工程。办好珠海城市职业技术学院，培养适应现代制造业和服务业发展需要的技能型人才。继续办好大学园区，引导高校与珠海经济和社会互动发展。引导规范社会力量投资办学，促进民办教育发展。推动文化盛市建设。加快文化事业和文化产业发展，抓好文化市场管理。完成图书馆装修工程和搬迁，加快广播电视发射台、古元艺术馆和基层文化站等文化工程建设，重视群众文化场所的规划建设。继续加强人文历史资源保护。做好人才工作，加强人才区域合作和海外智力引进，营造有利于各类人才创业、工作、生活的社会环境。切实加强公共卫生体系建设，提高应对突发公共卫生事件的能力。加快市人民医院、拱北医院、市妇幼保健院改扩建工程。改革卫生事业投入体制，加快改造薄弱乡镇卫生院。发展体育事业和体育产业，办好国际跳水（中国站）、国际汽联GT世界锦标赛（中国站）等重大赛事。继续做好人口与计划生育工作，稳定低生育水平。继续贯彻男女平等基本国策，实施妇女儿童发展规划，维护妇女儿童合法权益。加强国防教育，加大民兵预备役和国防动员工作力度，继续做好双拥工作。继续做好民族、宗教、人防、国安、对台、外事、侨务、仲裁、统计、档案、气象、工会、老年人、残疾人和港澳流动渔民等工作，支持各人民团体按照职责开展相关工作。

　　为民多办实事。扎实推进省委、省政府确定的“十项民心工程”。重点办好公共设施建设和服务于人民群众生产生活的一批实事，主要包括：转移一万名农村富余劳动力到第二、第三产业就业，提高转移就业质量；实施让一万名珠海残疾人受惠的“助残工程”；建设西坑尾垃圾填埋场；建设前山沿河东路南段；完善路灯、公共汽车候车亭等道路配套设施；加快平沙、红旗危房改造；新建市计划生育服务站；建设安居园和新村两所九年一贯制学校；新建市社会福利中心；建设全市安全生产事故应急救援指挥中心等。各区、镇、街道办都要结合本辖区的实际，为人民群众多办实事。

　　五、优化环境，增强可持续发展能力

　　突出规划的龙头地位。认真实施《珠江三角洲城镇群协调发展规划（2004-2020）》和《珠海市城市总体规划（2001-2020）》，坚持以规划指导可持续发展。今年要落实规划经费2000万元，加快编制各层次的城市规划，重点开展城市发展策略研究和编制近期建设规划。规划编制要充分发挥城市规划委员会和专业技术委员会的作用，建设城市规划信息管理体系，鼓励公众参与。理顺规划管理体制，加强执法监督，维护规划的权威性，规划一旦经法定程序批准就必须严格执行。

　　加强环境保护和生态建设。要倍加珍惜珠海的环境优势，把加强环境保护和生态建设摆到更加重要的位置来抓。大型项目环境影响评估工作要接受公众监督。大力实施治污保洁工程，加快金湾区和斗门区污水管网、污水处理设施建设，对前山河等重点区域、重点污染源头进行综合整治，着力解决威胁群众生命健康安全的环境污染问题。研究制订机动车尾气污染防治管理办法。加强湿地和生物物种资源保护管理。

　　优化城市环境。加快理顺城市管理体制，完善相对集中行使行政处罚权执法制度，增强执法效能。推进城市管理综合整治，积极探索堵疏结合、标本兼治的新路子，提升城市管理水平。制定政策鼓励盘活烂尾楼，治理违规改变建筑物使用功能行为。进一步完善市政配套设施，提高现有市政设施管养水平。提高绿化覆盖率，建设林荫大道，营造和维护优美的城市环境。

　　发展循环经济。认真落实国家和省关于开展资源节约活动的一系列工作部署，制订实施节能降耗与资源综合利用政策、法规和标准，大力推行清洁生产。加强宣传教育，引导公众积极参与建设节约型社会。普及节能节水技术，鼓励高耗能、高耗水企业进行节约资源技术改造，提高土地、水、能源和重要原材料的综合利用效率。逐步引入绿色GDP考核体系，推动经济增长方式转变。

　　保护海岛和海洋资源。落实珠海市海洋功能区划。认真实施《广东省人工鱼礁管理规定》和《珠海市珊瑚资源管理办法》，抓好人工鱼礁建设、利用和保护管理，增殖渔业资源。加强岸线保护和出海口流域、近海海域环境综合治理，保护和改善海洋生态环境。

　　六、依法行政，不断加强政府自身建设

　　加快推进政府职能转变。深入贯彻《行政许可法》和国务院《全面推进依法行政实施纲要》，深化行政管理体制改革，加快职能转变，在继续搞好经济调节、加强市场监管的同时，强化政府的社会管理和公共服务职能。建立健全各种预警和应急机制，提高保障公共安全和处置突发事件的能力。

　　提高科学行政、民主行政、依法行政能力。遵循客观规律，推进政府决策科学化、民主化、法制化。重大决策要慎之又慎，要对人民负责，重大事项通过听证等方式广泛征求意见，避免出现重大失误，避免出现短期行为。自觉接受监督，定期向市人大及其常委会报告工作，认真执行市人大决议。积极支持人民政协履行政治协商、民主监督、参政议政职能，主动听取各方面对政府决策的意见。规范政府行为，严格按照法定权限和程序行使权力、履行职责，做到有权必有责、用权受监督、侵权须赔偿、违法要追究。加大政务公开力度，增强政府工作的透明度，让人民来监督政府，政府工作人员才会永不懈怠。

　　加强政风建设。开展“理想、责任、能力、形象”教育活动，加强公务员培训，积极创建学习型组织。继续加强机关作风建设，大力弘扬求真务实的精神，深入基层，贴近群众，力戒作风飘浮和形式主义、文牍主义。继续加强督查督办，狠抓各项决策的落实。稳妥推进公务车改革，节约行政成本。切实加大行政监察力度，坚决纠正损害群众利益的不正之风，落实廉政建设各项措施，构筑教育、制度、监督并重的惩治和预防腐败体系，努力建设为民、尽责、务实、廉洁和人民满意的法治政府。

　　做好“十一五”规划编制工作。以科学发展观为指导，用改革的精神、创新的思路、科学的方法编制“十一五”规划，努力使“十一五”规划充分体现以人为本、全面协调可持续发展的理念，更好地指导珠海今后五年经济和社会发展。

　　各位代表！2005年是充满希望与挑战的一年。我们要在邓小平理论和“三个代表”重要思想指引下，紧密团结在以胡锦涛同志为总书记的党中央周围，在省委、省政府和市委的正确领导下，在市人大、市政协的监督支持下，团结带领全市人民努力践行科学发展观，与时俱进，开拓进取，扎实工作，为实现珠海发展的美好蓝图而努力奋斗！
